# Climate change and its impact on health: a global collaborative learning model

**DOI:** 10.3389/fmed.2024.1438609

**Published:** 2024-08-16

**Authors:** Harriet Okatch, Patricia A. Remshifski, Anita Fennessey, Haley Campbell, Sivia Barnoy, Jason Friedman, Stephen B. Kern, Rosemary Frasso, Cecilia Sorensen, Tami Bar-Shalita, Louis N. Hunter

**Affiliations:** ^1^Jefferson College of Population Health, Thomas Jefferson University, Philadelphia, PA, United States; ^2^Department of Speech-Language Pathology, Jefferson College of Rehabilitation Sciences, Thomas Jefferson University, Philadelphia, PA, United States; ^3^Jefferson College of Nursing, Thomas Jefferson University, Philadelphia, PA, United States; ^4^Department of Environmental Health Sciences, Mailman School of Public Health, Columbia University, New York, NY, United States; ^5^Global Consortium of Climate Change and Health Education, Mailman School of Public Health, Columbia University, New York, NY, United States; ^6^Department of Nursing, School of Health Professions, Faculty of Medical & Health Sciences, Tel Aviv University, Tel Aviv, Israel; ^7^Department of Physical Therapy, School of Health Professions, Faculty of Medical & Health Sciences, Tel Aviv University, Tel Aviv, Israel; ^8^Department of Occupational Therapy, Jefferson College of Rehabilitation Sciences, Thomas Jefferson University, Philadelphia, PA, United States; ^9^Asano-Gonnella Center for Research in Medical Education and Health Care, Sidney Kimmel Medical College, Thomas Jefferson University, Philadelphia, PA, United States; ^10^Department of Emergency Medicine, Columbia University, New York, NY, United States; ^11^Department of Occupational Therapy, School of Health Professions, Faculty of Medical & Health Sciences, Tel Aviv University, Tel Aviv, Israel; ^12^Jefferson College of Health Professions, Thomas Jefferson University, Philadelphia, PA, United States; ^13^Department of Physical Therapy, Jefferson College of Rehabilitation Sciences, Thomas Jefferson University, Philadelphia, PA, United States

**Keywords:** climate change, interprofessional, healthcare, curriculum, international, course development, symposium

## Abstract

To address the health effects of climate change, leaders in healthcare have called for action to integrate climate adaptation and mitigation into training programs for health professionals. However, current educators may not possess sufficient climate literacy and the expertise to effectively include such content in their respective healthcare curricula. We, an international and interprofessional partnership, collaborated with experts to develop and deploy curriculum to increase health educators’ and graduate health profession students’ knowledge and competencies on climate change. In a tri-step process, the first phase included recruiting interested faculty members from two institutions and varying health professions. In phase two, faculty members collaborated to develop a faculty symposium on climate change including educational competencies required of health professions, practice standards, guidelines, and profession-specific content. Symposium outcomes included broader faculty member interest and commitment to create an interprofessional climate change course for healthcare graduate students. In phase three, course development resulted from collaboration between faculty members at the two institutions and faculty members from the Global Consortium on Climate and Health Education (GCCHE), with course objectives informed by GCCHE competencies. Climate experts and faculty members delivered the course content over a 10-week period to 30 faculty members and students representing seven health professions, who were surveyed (n = 13) for feedback. This course can serve as an example for international collaborators interested in developing climate change courses for health profession students. Lessons learned in this process include: climate change novice faculty members can develop impactful climate change courses; students and faculty members can be co-learners; diverse representation in course attendees enriches the learning experience; and collaboration is key.

## Introduction

The World Health Organization (WHO) and the Intergovernmental Panel on Climate Change have highlighted the direct negative impact of climate change on the environmental determinants of health, including sufficient safe water, food and shelter (1, 2). These climate-related events are accompanied by myriad adverse health effects that can overwhelm healthcare systems and pose challenges to health practitioners who may not have had training in preventing, diagnosing and treating climate-related health. The WHO predicts that climate change will cause approximately 250,000 additional deaths per year between 2030 and 2050 (1). Since climate change is associated with increases in morbidity from infectious, cardiovascular, respiratory and neurological diseases (3), leaders in healthcare have called for action to integrate climate adaptation and mitigation into health professions education (HPE) (4–7).

Although the climate crisis demands that today’s health professionals understand its effects on health and implications for treatment (1, 8), healthcare providers are, however, not trained or equipped to address climate change impacts but have reported interest in developing the appropriate knowledge and skills (9–11). Globally, however, climate change education is generally not mainstream or mandatory, hence current health professional students may not graduate with the skills needed to address climate change in their future roles (8, 12). Health professionals may not intuitively envision their role in climate change education and advocacy for policies that would protect their patients/clients. This may be because the educators themselves are not proficient in climate change issues since most professional educational standards do not currently require that curriculum include climate change (13).

Existing climate change and health curricula have utilized varying design approaches and have been developed for specific audiences (14–16). Recognizing that faculty members may have time constraints with heavy teaching responsibilities, established research agendas in areas independent of climate change, and limited climate expertise, we utilized a global collaborative model to develop a climate change course that places faculty members both as educators and learners. The target audience for this pilot course included both faculty members and students from a range of health professions. Here, we describe the processes undertaken by a team of interdisciplinary educators from three institutions in two countries to increase climate literacy for health professions faculty members and students.

### Increasing climate literacy

**Figure 1:**
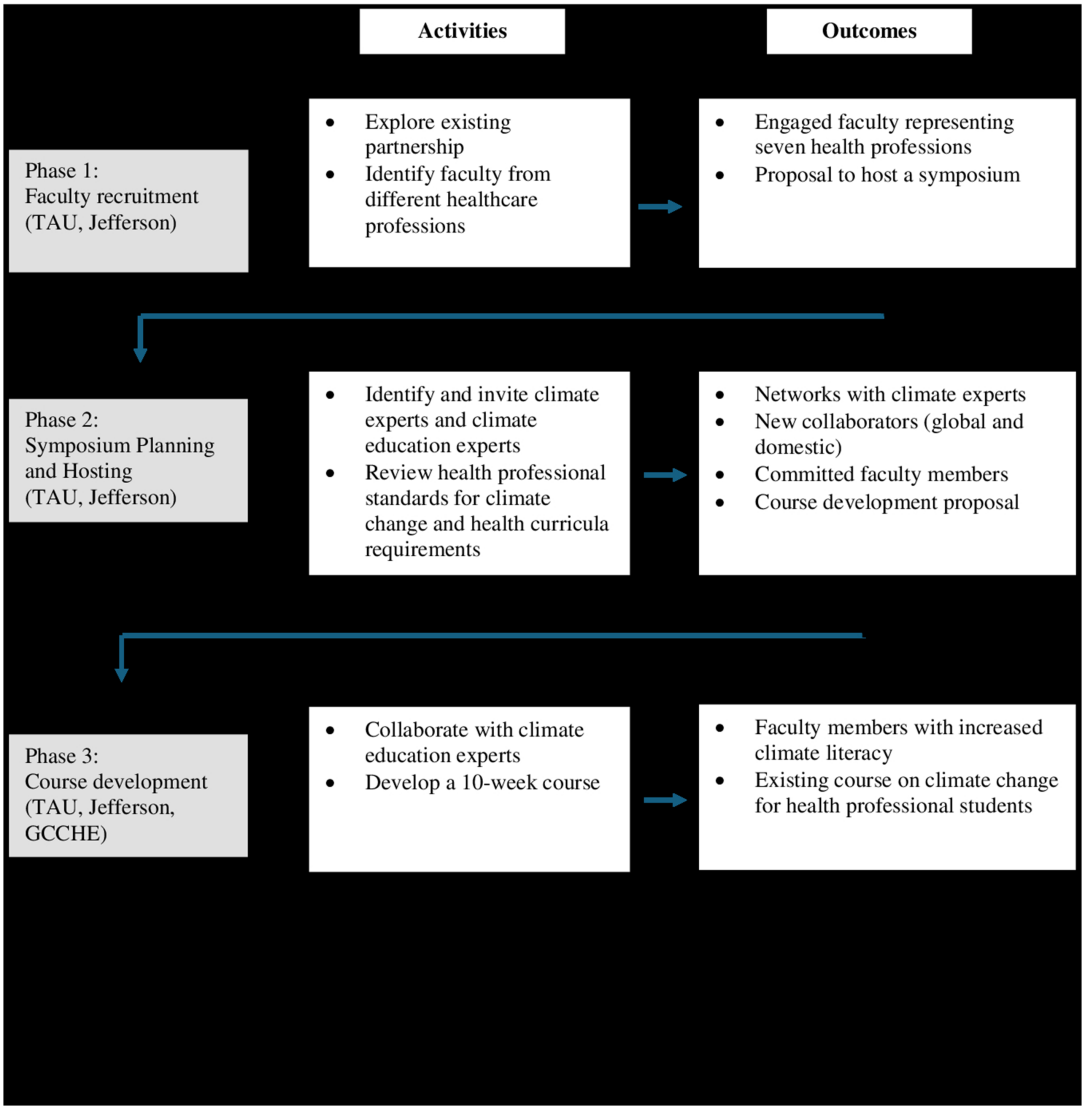
A summary of the phases, activities and outcomes for increasing climate literacy among faculty members and students in health disciplines. TAU, Tel Aviv University; Jefferson, Thomas Jefferson University; GCCHE, Global Consortium on Climate and Health Education.

A triphasic process, described below, was used to increase climate literacy among faculty members. Figure 1 summarizes the phases, activities, and outcomes involved in this unique model for increasing climate literacy for both faculty members and students.

#### Approach

##### Phase 1: Faculty member recruitment

In 2018, The Jefferson Israel Center was established between Tel Aviv University (TAU), Israel, and Thomas Jefferson University (Jefferson), United States, with the goal to partner in academic and clinical research. Through this existing partnership and several meetings, faculty members identified two major priorities: Develop bilateral student exchange and co-host a virtual event. These initial meetings allowed faculty members to meet, present their areas of research interests and current work, and identify an area of interest to collaboratively research.

Faculty members from both academic institutions attending the planning meetings represented Occupational Therapy, Physical Therapy, Speech-Language Pathology, and Nursing. They decided to focus academic and research collaboration on the neglected area of climate change and natural disaster management from an interprofessional perspective. The final group of faculty stakeholders comprised healthcare faculty members who were interested and committed to incorporating climate change and health into graduate (Masters) HPE.

##### Phase 2: Symposium on climate change and health education: planning and hosting

Faculty members from the two academic institutions collaborated to plan a global educational symposium as an interprofessional opportunity for participants to learn about impact of climate change and climate-related disasters on health, especially for vulnerable populations. This educational symposium was entitled “Educational Symposium on Climate Change 2022: An Introduction to the Role of the Healthcare Professional during Climate Change Events.” It took place in late May at a mutually acceptable time for US and Israeli participants. It was held in-person at each institution as well as virtually. The aim of this symposium was to increase awareness and understanding of the impact of climate change on health for attendees. It also provided an opportunity for various health professionals to consider how to address climate change from their profession’s perspective and through the lens of curriculum development and accreditation standards.

Symposium presentations comprised,

- Expert lecture: Globally recognized climate experts delivered presentations aimed at either increasing climate literacy or exploring gaps in climate-related content in curricula. Climate experts presented on the role and impact of climate change on health, and advocacy in clinical practice.
- Panel: To identify opportunities to include climate and health content, a panel comprising of faculty members from the Nursing, Occupational Therapy, Speech-Language Pathology, Medical Laboratory Sciences and Biotechnology, and Community and Trauma Counseling provided an overview of each profession’s current programs and the extent to which climate change had been included into curriculum. In this process, the faculty panel identified and described each health profession’s official statement on climate change and sustainability regarding inclusion of climate change in the curriculum. Faculty members included examples of how each health profession could commit to the inclusion of climate change in their respective curricula.
- Climate change competencies: Representatives from the Global Consortium on Climate and Health Education (GCCHE), housed at the Joseph L. Mailman School of Public Health at Columbia University, described their efforts to educate health professionals specifically addressing the *Climate & Health Core Concepts.* The *Core Concepts* are a highly vetted set of global educational standards that serve as a guide for HPE. This document covers climate and health analytic skills and knowledge, communication and collaboration, policy, and public health as well as clinical practice competencies (17).

Feedback and outcomes: During the debrief session, faculty members acknowledged that several perceived outcomes were attained during this phase. They recognized increased knowledge on climate change and the associated health effects. In addition, they appreciated the formation of new connections with the symposium presenters, recognizing these climate experts as a resource for future potential collaboration. Finally, having recognized the dearth of climate content in health professions standards, faculty members were motivated to develop a collaborative course for graduate health professions students focusing on the respective profession’s role in addressing the negative health impacts of climate change, specifically for vulnerable populations.

##### Phase 3: Course development

Faculty members from the GCCHE were invited to co-develop an international course for graduate health professions students, with faculty members serving as learners and facilitators.

Throughout all phases, institutional TAU and Jefferson leadership was supportive of the need to explore climate change and HPE. Both TAU and Jefferson provided funding for this research, including the educational symposium and the course. The next section will describe the course development.

## Competencies and pedagogical frameworks guiding the course development

### Global consortium on climate and health education competencies

The Climate & Health Core Concepts were used as guiding principles in developing this course (Box 1) (17). These concepts are a living document designed to be flexible to incorporate emerging science, yet stable to allow thoughtful curricular planning. This framework is intended as a blueprint for developing climate and health education within health professional schools as well as in continuing education programs for practicing health professionals. This framework consists of Domains (categories of educational activities), Concepts (overarching principles that form the foundation of climate and health knowledge and skills) and Learning Objectives (a brief statement that describes what students can be expected to do after successful learning relating to a concept), which can be applied and integrated as needed (17). The Climate & Health Core Concepts for Health Professionals provided the educational foundation for this course and were tailored accordingly to accommodate the requirements of the institutional partners and meet the needs of the participating international and interprofessional graduate student audience.

BOX 1 **The climate and health core concepts and competencies created by the Global Consortium for Climate and Health (GCCHE) (17).**

- Define climate drivers (both natural and human-caused), weather, climate change, and climate variability.
- Demonstrate understanding of the scientific consensus on climate change and concept of evolving science.
- Applies fundamental knowledge of ecology, biology, and complex systems in environmental science.
- Apply knowledge of levels of prevention, climate mitigation and adaptation, and explain health co-benefits of actions.
- Describe public health and its determinants.
- Access and interpret relevant local, regional, national, and global information about climate change effects on health.
- Apply knowledge of the ethical, professional, and legal obligations relevant to climate and health.
- Identify the health impacts of climate change and effective responses on the part of specific health services.
- Apply knowledge of emergency planning skills.
- Demonstrate effective communication with stakeholders about climate and health topics.

### Transformative learning theory

Introduced by Mezirow, the Transformative Learning Theory (TLT) is a process that involves several progressive stages, starting with a disorientating dilemma where pre-existing perceptions are disrupted, followed by critical examination of assumptions, then recognition that others are experiencing a similar reckoning; it leads learners to explore options for new roles, relationships, and actions (18). TLT includes a process by which learners develop a new frame of reference in their perception of their lives and environment and affords learners freedom to explore issues “beyond the formal curriculum, such as social justice” (19).

Transformative learning can be explained as a form of metacognitive reasoning. Reasoning is the process of advancing and assessing explanations, especially those that provide points of view supporting beliefs resulting in decisions to act. Beliefs are justified when they are based on good rationale. The process of cognitive reasoning may involve such concepts as aptitudes, skills, and competencies (20). Appropriate elements of TLT, such as discourse and communicative learning, were incorporated into the course. TLT served as the guide for team reflection and evaluation of this course for the faculty members.

TLT Learning occurs as individuals engage in the process of critical reflection and analysis of their underlying assumptions. Frequently, healthcare educators function as “coaches,” facilitating and guiding reflection and analysis through this critical discourse (21). This engagement and empowerment of learners results in the development of a plan for action, and the acquisition of the knowledge to implement the developed plans. Typically, learners engage in role play to gain confidence and competence to integrate the newly acquired information and skills into their personal and professional lives. By design, this interprofessional course engaged learners in group activities. This was done to facilitate new communities of learning and development of leaners’ professional identities. Ong et al. explored shifts of learners’ perspectives attributed to TLT as “new ideas, practices, or approaches,” “new insights into existing concepts,” and “new relationships [that] foster critical discourse for learning” (22).

## Learning environment (setting, students, faculty members); learning objectives; pedagogical format

The learning objectives in Table 1 were modified from the GCCHE competencies to increase climate change knowledge, communication, and advocacy. The chosen objectives were designed to present the impact of climate change from an international and interprofessional perspective that includes health impacts; health policy; global and regional risks; and role identification of various stakeholders engaged in climate and health action.

**Table 1:** The 10 learning objectives of the course, modified from the GCCHE competencies (17).

#	Learning outcome
1	Identify the health impacts of climate change and effective responses on the part of specific health services.
2	Apply knowledge of levels of prevention, climate adaptation, disaster responses and explain health co-benefits of climate actions.
3	Identify the risks and vulnerabilities to critical health infrastructure impacted from climate changes.
4	Use emergency planning skills to plan for and respond to climate-related extreme weather events and disasters, including workforce surge needs, and distinguish the roles of and interactions between agencies involved in emergency care.
5	Explain the role of local, regional, national and global policy frameworks and governance structures to address health risks associated with climate change and natural human disasters.
6	Apply climate and health knowledge to improve decisions about health services and impact on improving population health.
7	Access and interpret relevant local, regional, national and global information about the effects on health resulting from climate changes and disaster occurrences.
8	Apply knowledge of the ethical, professional, and legal obligations relevant to climate and health.
9	Describe the roles and responsibilities of the different health professions in preparing and responding to the climate-related health crisis.
10	Foster global cooperation and convergence on climate adaptation and mitigation.

The “International and Interprofessional Perspectives on the Impact of Climate Change & Climate-Related Disasters on Health and Health Care Delivery” course was delivered online over 10 weeks and included the following topics: Climate Change for the Health Professional; Extreme Weather Hazards; Climate Change and Health Equity; Food Security; Degraded Air Quality; Temperature-Related Illness and Mortality; Climate and Health Communication; and Health Sector Mitigation. Each topic was framed using the United Nations (UN) 2030 Sustainable Development Goal 13 “Climate Action.” According to the UN, “Climate Action” requires “urgent action to combat climate change and its impact.” Health professionals are on the front lines of mitigating the negative health impacts of climate change, and education is the first step in any change or action (23).

The course involved synchronous and asynchronous learning experiences using an interprofessional healthcare approach. International experts provided synchronous lectures and learning opportunities. Both individual-level assignments and group work were included in the course. The assignments were asynchronous and included quizzes, reflection critiques, and a final examination. Two types of group assignments were included; weekly case-based problems which were completed during the synchronous sessions, and a final project that the students worked on asynchronously over the duration of the course. All assignments with grading rubrics were created and approved by the academic institutions involved in this collaboration. The GCCHE at Columbia University had previous course development expertise and recruited the speakers as part of its consultant role, so it was important for consistency that the GCCHE’s Project Director grade all assignments. A survey was administered at the end of the course to capture student and faculty member feedback.

## Results

### Course details

Each weekly 90-min synchronous session included a 45-min lecture delivered by a climate change expert, followed by a 10-min Q&A session (Table 2). A 30-min discussion facilitated by a faculty member then followed during which students worked in groups of four to six on a climate change case study. In these peer-led groups, students collaboratively analyzed clinical scenarios, synthesized information from different sources, and were encouraged to think in new ways. An emphasis was placed on exploring different perspectives as well as on generating alternative approaches to practice challenges and emerging opportunities.

**Table 2:** Example of the activities and the allocated times in the 90-min synchronous sessions.

Time	Activity	Description
45 min	Lecture	Topic: Degraded Air Quality
10 min	Q & A	Students and faculty members had the opportunity to ask the speaker questions related to the lecture content.
30 min	Group work	Students were split in five groups of four to respond in discussion to the prompt below. The discussions were facilitated by a faculty member. Discussion prompt: *Your hospital’s executives have asked you to give a presentation regarding air quality, health, and climate change. They have been resistant to the idea of adopting climate-smart policies in their healthcare system because they have yet to see how climate change can impact health or hospital expenses. However, after two straight weeks of terrible air quality from wildfires during the fall, they are concerned about the level of respiratory admissions which are stretching their hospital resources very thin.* *As the hospital’s climate and health specialist, you are tasked with educating the executives about climate change and poor air quality and how it impacts health. Unfortunately, they gave you short notice to compile your presentation with your team. Work with your team to craft a brief presentation regarding climate change, air quality, and health.*
5 min	Wider group discussion	This time was used to succinctly share points discussed in the small group discussion with the wider group.

Besides the weekly group work, students were assigned to groups in which they developed a project focusing on the relationship between climate change and health outcomes. Each group chose an area of interest from the course topics and prepared a presentation responding to the prompts focusing on their selected topic. Each group presented their findings to the course participants during the last 2 weeks of the course. Students worked asynchronously for 2.5 weeks to complete the assignment.

The focus of the presentations included health professionals’ responses to known health impacts of a changing climate. Students were instructed to approach the issue with an interdisciplinary and international lens, describe how different health professions can collaborate, and identify how individual health practitioners can be mobilized to respond. The prompt for the final project is shown below.

*Identify a specific health issue related to a climate exposure. Through collaborative research with your peers, answer the following questions:*

1. *Introduction: Why is this topic important and relevant NOW? Provide background on the specific climate related health threat.*
2. *Problem statement: What health challenges need to be addressed?*
3. *Climate attribution: How climate change and related exposure pathways are impacting the problem?*
4. *How can health professionals respond to the health impacts? What has been effective? Approach the issue with an interdisciplinary lens.*
5. *Further directions: What information or research is needed to improve our understanding of the issue to guide a successful response from health professionals globally.*

### Course participant recruitment

Recruitment of TAU students involved sending emails to all students studying for a Master’s degree in various health professions (Occupational Therapy, Communication Disorders, Physical Therapy, Nursing, and Emergency and Disaster Medicine). TAU faculty members also attended various classes to present the course and encourage enrolment. Recruitment of students at Jefferson involved similar processes but also included completion of an application form following advertisement. Jefferson students were enrolled from the following health professions: Physical Therapy, Nursing, Speech Language Pathology, Public Health, Occupational Therapy, and Biotechnology.

### Course attendance

Faculty members who planned the symposium and developed the course also participated in the class sessions and facilitated discussions. These faculty members selected the classes they would attend and facilitate based on their interest.

The pilot course was restricted to 30 participants. Twenty students and 10 faculty members representing seven health professions participated in this course. TAU students were in Physical Therapy (*n* = 5), Occupational Therapy (*n* = 4), Nursing (*n* = 2), and Disaster management (*n* = 1) and Jefferson students were in Physical Therapy (*n* = 1), Occupational Therapy (*n* = 2), Public Health (*n* = 4), and Biotechnology (*n* = 1). The faculty members from TAU were from the following programs Physical Therapy (*n* = 1), Occupational Therapy (*n* = 2), Nursing (*n* = 1) and those from Jefferson were from Physical Therapy (*n* = 1), Occupational Therapy (*n* = 1), Nursing (*n* = 1), Public Health (*n* = 2) and Speech-Language Pathology (*n* = 1). All faculty members were involved in all aspects of course development.

### Course evaluation

Of the 20 students and 10 faculty members who participated in the course and given the same set of evaluation questions, 13 (43%) completed the survey. Based on the feedback, the speakers were highly rated with mean scores greater than 3.5/4 on the following metrics: Knowledgeable, provision of current evidence, effective delivery, appropriate tone of voice and body language, and responding to questions. Overall student and faculty members agreed that the course objectives were well connected to the learning objectives, and they valued the course as having an impact on their education and practice in terms of climate literacy (Table 3).

**Table 3:** Student and faculty members’ feedback on course format, content, and delivery.

Feedback categories	Response summary and selected quotes
Course format and delivery	Weekly didactic lectures followed with group activities and discussions was positively noted as a good format. Generally, the expert lectures were found to be engaging and interesting. The virtual format of the course was appreciated as students could review the video recordings asynchronously at their own pace. “Most of the lecturers were good and very interesting.” “It was online and recorded (I did join in most classes but could review them again and make up for the ones I could not attend)” “Weekly group activities and discussions with students and faculty.”
International perspective	Interactions with international students were highly valued, offering diverse perspectives, insights into different social support systems and discussion of each country’s unique climate change response. “The final group project allowed for interprofessional and international collaboration to explore and present on a specific climate change topic impacting health.” However, some respondents noted that there was insufficient information on the impact of climate change on health in various countries. “The guest speakers could have benefitted from including more data and information about the impact of the various climate change topics on health in Israel and other countries other than the U.S., especially those vulnerable and/or developing.”
Interprofessional collaboration	Respondents generally reported an appreciation for the interprofessional collaboration opportunities provided by the course. Students enjoyed engaging with individuals outside their field of study through discussion and group work. “Being able to talk and engage with those outside of my field of study.”
Guest speakers’ presentation focus	It was suggested that an emphasis was needed on implementable healthcare action steps related to climate change. “... more of their (speakers) allocated time to present could have been dedicated to implementable healthcare action steps and our role in combatting the discussed climate change topics.”
Pace and content of some speakers	The respondents expressed desire for more direct connection to specific professional roles and actionable steps related to each climate and health topic. It was noted that some presented spoke too quickly for an international audience to follow the content. Some individuals remarked on the unequal distribution of assignment responsibilities, particularly noting disparities between students enrolled for credit and those voluntarily participating. “Most speakers were fine. Some talked too fast. Most talk more about the problems and less about solutions.”

## Discussion on the practical implications and lessons learned

As a group of 10 educators from a range of health professions, we successfully collaborated to develop a course that positioned us as learners while simultaneously training 20 health professional students from two countries. While this model for educating health professionals on climate change has not been previously described, it does align with TLT in that both faculty members and students developed an expanded interprofessional frame of reference in their perception of the impact of climate change on health. Descriptions of climate change courses for health professionals have either enrolled one discipline of health professionals (e.g., medical students only) (14), have been offered to only health professionals in practice (24), or are offered exclusively as asynchronous (15). Some of the described courses have similar elements to our course; however, none of them include faculty members and students as simultaneous learners in a course developed by faculty members and offered across two institutions. While Rom (16) developed a course that has now been offered at three institutions sequentially, our course can be offered at multiple institutions simultaneously.

Through this course development and deployment, faculty members gained knowledge in climate change and health while simultaneously educating health professional students. This example of student and faculty member co-learning can serve as a model to rapidly deploy HPE in response to emerging health crises, including the climate crisis and the remaining triple planetary crises of biodiversity loss and pollution.

### Lessons learned

#### Climate change expertise not required

Climate change *expertise* is not a pre-requisite for developing a course that is impactful. None of the educators who collaborated on this course was a climate expert. Notwithstanding, they successfully invited expert guest lecturers and facilitated learning through guided discussions. Each faculty member was sensitive to climate change impacts and therefore open to collaborating and learning. It is therefore important to strategically identify such faculty members who will remain committed to the process of educating themselves and others through course development and delivery. Our results suggest that climate change novices can creatively design courses for students even though they currently do not possess the appropriate knowledge. As suggested by Shaw et al., the climate crisis calls for change in how education is approached (25). Faculty members who are experts at sharing wisdom with learners in their own specific fields need to stand side by side with their colleagues and their students to create space to learn and collectively respond to crises.

#### Institutional collaboration is key

Collaboration through existing or novel partnerships was instrumental to the success of this course. In this case, an existing partnership between the two institutions provided the foundation for collaboration. The current impact of climate change on health and the environment has potentially generated concern among several faculty members to think about it. An invitation to partner on a collaborative course development can be the motivator for faculty members to contribute to climate change course development.

The symposium and the course provided faculty members with additional networking opportunities with climate experts who presented at the symposium or delivered the lectures. Moreover, the materials they presented or shared become resources that faculty members and students could utilize to develop climate literacy. Further, in the learning process, student collaborations led to an enriched learning experience for the students.

#### Faculty members and students can learn simultaneously

In our course, faculty members and students were simultaneously enrolled as learners in the course. All learners attended the course content delivered by the climate experts. The discussion sessions allowed for learning from one another. However, the faculty members did not have to complete any of the assignments. Faculty members increased their climate proficiency, equipping them to then develop more institution-specific climate change courses. Students were able to observe their faculty members as learners and were exposed to different, and possibly more advanced, learning and knowledge processing styles.

#### Diversity enhances learning

The climate experts who presented at the symposium and during the course originated from a range of professions, institutions, and countries, allowing learners to gain different location-and profession-specific perspectives, thereby enriching the learning. Climate change is a universal experience, and global co-operative learning can provide faculty members and students the opportunity to understand more realistically how their actions impact others, learn about different mitigation and adaptation strategies, and collectively develop solutions. The course participants (faculty members and students) who were also diverse represented various health professions from two institutions.

### Pragmatic considerations when developing international coursework

#### Institutional differences

Collaborating faculty members may need to review university-level curricula development guidelines and their respective curriculum committee guidelines as this can direct the course development process. In our case, one institution was unable to offer the course to its students as a credit-bearing course due to the institution structure, specification, and time required to approve new courses. Furthermore, student recruitment may be specific to the respective institutional guidelines.

Another barrier was that the two institutions used different education platforms for course content management. To overcome this, a common location was created. Interested faculty members might want to check their institution-specific guidelines about shared document management software.

Notwithstanding, such institutional bureaucracies should not dampen collaborative efforts to develop inter-institutional climate change courses. In fact, offering this course simultaneously as a credit course at one institution and as non-credit at the other institution demonstrates flexibility of the course design.

#### Logistics

Working internationally presents several logistical considerations. A logistical challenge was the six-hour time difference that provided a narrow window of time during which the course could be offered. Faculty members at other institutions who might want to adopt this model may need to be creative in terms of timing and/or format of course delivery to accommodate time differences. Despite this logistical challenge, the successful launch and delivery of the course demonstrated the ability to synchronously offer an international online course with learners from different places.

#### Pedagogical considerations

Because of the predicted diverse groups of health professions students anticipated to enroll in the course, we chose to utilize a co-operative learning format. The small groups were deliberately diverse with respect to profession and country of residence, but also in terms of race and academic background. Furthermore, we chose group work because it is associated with more active learning; knowledge development, which include critical thinking, time management and leadership, refined idea articulation, as well as a knowledge of the topic. The ability to have critical discussions in small groups is an important part of transformative learning (21, 26). Small groups require participants to share their thoughts and opinions, which is an important part of the learning process. In small groups, students cannot remain passive, and by being part of the conversation, they are encouraged to critically examine and interact with the ideas being presented, increasing the chance of broadening and deepening their understanding.

### Reflections and next steps

Based on the student and faculty members’ feedback, we plan to further develop the course to offer additional content on Policy, Public Health Practice, and Clinical Practice. This correlates with the imperative that healthcare practitioners need sufficient foundational knowledge to understand the detrimental effects of climate change on health as well as the need for interprofessional collaboration among stakeholders to improve health outcomes. Most participants had not made a connection between understanding what climate change is and connecting it to poor health outcomes. For example, there are increased incidences of heat waves and floods (2), but participants may not make the connection. Therefore, future iterations of this course will expand the scope, including the importance of health professionals understanding how health policies may improve or exacerbate health impacts. In addition, future iterations will also integrate the impact of public health practices in addressing the impacts of climate change from a prevention perspective. Examples include developing effective public health warnings about how to stay cool during heat waves, and strategies to mitigate emission in the health sector.

Furthermore, we deliberated upon two next steps: initially, to extend the offering of the developed curriculum in its current state with newly forged global partnerships, or as an in-person, week-long academic exchange within a carefully chosen venue. This iteration of the course would incorporate the existing recorded lectures as well as enlist identified speakers to record lectures for dissemination among the students. These pre-recorded lectures can then be accessed asynchronously, allowing for increased engagement in active learning pertaining to the role of health professionals within relevant contexts. Additionally, experiential learning will encompass visits to healthcare facilities, facilitating an understanding of the respective challenges in healthcare delivery within the framework of climate change.

Secondly, aiming to furnish a blueprint or offer ideas for individuals who are motivated to run similar courses but lack exposure to the development of such curricula, we have explored the prospect of organizing and hosting an international conference. We would extend invitations to individuals to share their experiences—both the opportunities and challenges—related to the development of climate change curriculum tailored for health professionals. Additionally, we envisage that this conference will attract a gamut of health professionals who may or may not have encountered climate change content yet within their educational curriculum. Furthermore, we anticipate the attendance of representatives from various organizations encompassing healthcare, public health, and related sectors, who have implemented strategies promoting climate change awareness, education, and advocacy specifically targeted at health professionals.

## Conclusion

This three-phase process to develop and then run a climate change pilot course by interprofessional faculty members from two institutions simultaneously increased climate literacy for faculty members and students. The course was built on the foundational competencies already established by the GCCHE and the collaboration provided access to climate change expertise and resources. The success of each phase (faculty member recruitment, symposium planning and hosting, and course development) was a result of collaboration, creativity, commitment and communication. The steps leading up to and including the developed course can be used as a model by climate novices to develop an international interprofessional collaborative climate change course for health professions education.

## Data Availability

The raw data supporting the conclusions of this article will be made available by the authors, without undue reservation.

## References

[1] World Health Organization . (2023). Climate change. Available at: https://www.who.int/news-room/fact-sheets/detail/climate-change-and-health (Accessed May 17, 2024)

[2] Intergovernmental Panel On Climate Change (Ipcc) . (2023). Climate change 2022 – Impacts, adaptation and vulnerability: Working group II contribution to the sixth assessment report of the intergovernmental panel on climate change. 1st ed. Cambridge University Press. Available at: https://www.cambridge.org/core/product/identifier/9781009325844/type/book [Accessed on May 23, 2024].

[3] Rocque RJ Beaudoin C Ndjaboue R Cameron L Poirier-Bergeron L Poulin-Rheault RA . Health effects of climate change: an overview of systematic reviews. BMJ Open. (2021) 11:e046333. doi: 10.1136/bmjopen-2020-046333, PMID: 34108165 PMC8191619

[4] Sherratt S . Communication and swallowing disorders: the effects of climate change. Perspect ASHA Spec Interest Groups. (2022) 7:245–58. doi: 10.1044/2021_PERSP-21-00186

[5] Cerceo E Saberi P Becker J. Interactive curriculum to teach medical students health and climate change. J Clim Change Health. (2022) 5:100105. doi: 10.1016/j.joclim.2021.100105

[6] World Federation of Occupational Therapy . (2018). Sustainability matters: Guiding principles for sustainability in occupational therapy practice, education and scholarship. Available at: https://wfot.org/resources/wfot-sustainability-guiding-principles (Accessed May 17, 2024)

[7] Wellbery C Sheffield P Timmireddy K Sarfaty M Teherani A Fallar R. It’s time for medical schools to introduce climate change into their curricula. Acad Med. (2018) 93:1774–7. doi: 10.1097/ACM.0000000000002368, PMID: 30024475 PMC6265068

[8] McKinnon S Breakey S Fanuele JR Kelly DE Eddy EZ Tarbet A . Roles of health professionals in addressing health consequences of climate change in interprofessional education: a scoping review. J Clim Change Health. (2022) 5:100086. doi: 10.1016/j.joclim.2021.100086

[9] Hathaway J Maibach EW. Health implications of climate change: a review of the literature about the perception of the public and health professionals. Curr Environ Health Rep. (2018) 5:197–204. doi: 10.1007/s40572-018-0190-3 29423661 PMC5876339

[10] Sarfaty M Kreslake JM Casale TB Maibach EW. Views of AAAAI members on climate change and health. J Allergy Clin Immunol Pract. (2016) 4:333–335.e26. doi: 10.1016/j.jaip.2015.09.018, PMID: 26703816

[11] Kotcher J Maibach E Miller J Campbell E Alqodmani L Maiero M . Views of health professionals on climate change and health: a multinational survey study. Lancet Planet Health. (2021) 5:e316–23. doi: 10.1016/S2542-5196(21)00053-X, PMID: 33838130 PMC8099728

[12] Tun May Sanyu Tun S Wellbery C Teherani A. Faculty development and partnership with students to integrate sustainable healthcare into health professions education. Med Teach. (2020) 42:1112–8. doi: 10.1080/0142159X.2020.1796950, PMID: 32762586

[13] Shea B Knowlton K Shaman J. Assessment of climate-health curricula at international health professions schools. JAMA Netw Open. (2020) 3:e206609. doi: 10.1001/jamanetworkopen.2020.6609 PMC7256668 32463471

[14] Aasheim ET Bhopal AS O’Brien K Lie AK Nakstad ER Andersen LF . Climate change and health: a 2-week course for medical students to inspire change. Lancet Planet Health. (2023) 7:e12–4. doi: 10.1016/S2542-5196(22)00304-7, PMID: 36608941 PMC9812426

[15] Rogers HH Tucker M Couig MP Svihla V. Facilitating an Interprofessional course on climate change and public health preparedness. Int J Environ Res Public Health. (2023) 20:5885. doi: 10.3390/ijerph20105885, PMID: 37239610 PMC10218104

[16] Rom WN . Annals of education: teaching climate change and global public health. Int J Environ Res Public Health. (2023) 21:41. doi: 10.3390/ijerph21010041, PMID: 38248506 PMC10815579

[17] Global Consortium on Climate and Health Education . (2023). Climate & Health Concepts for Health Professionals. Available at: https://www.publichealth.columbia.edu/file/11492/download?token=bBURLrFC (Accessed May 17, 2024)

[18] Mezirow J . Understanding transformation theory. Adult Educ Q. (1994) 44:222–32. doi: 10.1177/074171369404400403

[19] Van Schalkwyk SC Hafler J Brewer TF Maley MA Margolis C McNamee L . Transformative learning as pedagogy for the health professions: a scoping review. Med Educ. (2019) 53:547–58. doi: 10.1111/medu.13804, PMID: 30761602

[20] Briese P Evanson T Hanson D. Application of Mezirow’s transformative learning theory to simulation in healthcare education. Clin Simul Nurs. (2020) 48:64–7. doi: 10.1016/j.ecns.2020.08.006

[21] Vipler BS Sawatsky AP. When I say… transformative learning. Med Educ. (2023) 57:1184–6. doi: 10.1111/medu.15189, PMID: 37584372

[22] Ong CCP Foo YY Chiu FY Nestel D. ‘It’s going to change the way we train’: qualitative evaluation of a&nbsp;transformative faculty development workshop. Perspect Med Educ. (2021) 11:86–92. doi: 10.1007/S40037-021-00687-4, PMID: 34694570 PMC8543424

[23] United Nations . The 17 goals. United Nations; Available at: https://sdgs.un.org/goals (Accessed April 12, 2024)

[24] Sorensen C Hamacher N Campbell H Henry P Peart K De Freitas L . Climate and health capacity building for health professionals in the Caribbean: a pilot course. Front Public Health. (2023) 11:1077306. doi: 10.3389/fpubh.2023.1077306, PMID: 36778561 PMC9909391

[25] Shaw E Walpole S McLean M Alvarez-Nieto C Barna S Bazin K . AMEE consensus statement: planetary health and education for sustainable healthcare. Med Teach. (2021) 43:272–86. doi: 10.1080/0142159X.2020.1860207, PMID: 33602043

[26] Slavich GM Zimbardo PG. Transformational teaching: theoretical underpinnings, basic principles, and Core methods. Educ Psychol Rev. (2012) 24:569–608. doi: 10.1007/s10648-012-9199-6, PMID: 23162369 PMC3498956

